# Brain-Derived Neurotrophic Factor Val66Met is Associated with Variation in Cortical Structure in Healthy Aging Subjects

**DOI:** 10.14336/AD.2024.0346

**Published:** 2024-10-01

**Authors:** Ting Shen, Samran Sheriff, Yuyi You, Jiyang Jiang, Angela Schulz, Heather Francis, Mehdi Mirzaei, Danit Saks, Viswanthram Palanivel, Devaraj Basavarajappa, Nitin Chitranshi, Veer Gupta, Wei Wen, Perminder S. Sachdev, Huixun Jia, Xiaodong Sun, Stuart L. Graham, Vivek K. Gupta

**Affiliations:** ^1^Department of Ophthalmology, Shanghai General Hospital, Shanghai Jiao Tong University, School of Medicine, National Clinical Research Center for Eye Diseases, Shanghai Key Laboratory of Ocular Fundus Diseases, Shanghai Engineering Center for Precise Diagnosis and Treatment of Eye Diseases, Shanghai 200080, China.; ^2^Macquarie Medical School, Macquarie University, Sydney, NSW 2109, Australia.; ^3^Save Sight Institute, The University of Sydney, Sydney, NSW 2000, Australia.; ^4^Centre for Healthy Brain Ageing, School of Clinical Medicine, University of New South Wales, Sydney, NSW 2052, Australia.; ^5^Faculty of Health, Deakin University, VIC 3125, Australia.; ^6^Neuropsychiatric Institute, Prince of Wales Hospital, Randwick NSW 2031, Australia.; ^7^Neurology Department, Royal North Shore Hospital, St Leonards NSW 2065, Australia.

**Keywords:** brain-derived neurotrophic factor, Val66Met polymorphism, magnetic resonance imaging, aging, cortical morphology, entorhinal cortex, posterior cingulate cortex

## Abstract

Aging is associated with progressive brain atrophy and declines in learning and memory, often attributed to hippocampal or cortical deterioration. The role of brain-derived neurotrophic factor (BDNF) in modulating the structural and functional changes in the brain and visual system, particularly in relation to BDNF Val66Met polymorphism, remains underexplored. In this present cross-sectional observational study, we aimed to assess the effects of BDNF polymorphism on brain structural integrity, cognitive function, and visual pathway alterations. A total of 108 older individuals with no evidence of dementia and a mean (SD) age of 67.3 (9.1) years were recruited from the Optic Nerve Decline and Cognitive Change (ONDCC) study cohort. The BDNF Met allele carriage had a significant association with lower entorhinal cortex volume (6.7% lower compared to the Val/Val genotype, *P* = 0.02) and posterior cingulate volume (3.2% lower than the Val/Val group, *P* = 0.03), after adjusting for confounding factors including age, sex and estimated total intracranial volumes (eTIV). No significant associations were identified between the BDNF Val66Met genotype and other brain volumetric or diffusion measures, cognitive performances, or vision parameters except for temporal retinal nerve fibre layer thickness. Small but significant correlations were found between visual structural and functional, cognitive, and brain morphological metrics. Our findings suggest that carriage of BDNF Val66Met polymorphism is associated with lower entorhinal cortex and posterior cingulate volumes and may be involved in modulating the cortical morphology along the aging process.

## INTRODUCTION

Aging is characterised by genetic and epigenetic determinants leading to the progressive degeneration of endocrine, immunologic, and cognitive capabilities. This process is associated with increased brain atrophy, and learning and memory deficits, particularly due to hippocampal or cortical alterations [1]. The rise in life expectancy has correspondingly increased the incidence of cognitive decline and dementia, with older age being the primary risk factor for age-related pathologies, including Alzheimer's disease (AD) [2, 3].

Brain-derived neurotrophic factor (BDNF), which is well expressed in the hippocampus and cortex, is crucial for synaptic plasticity, neuronal survival, and differentiation [4, 5]. The loss of BDNF and its high-affinity receptor Tropomyosin receptor kinase B (TrkB) may contribute to hippocampal atrophy and memory impairment, which may be related to cognitive challenges experienced in AD [6]. The entorhinal cortex forms the anterior part of the parahippocampal gyrus and is a major source of inputs to the hippocampus [7]. It exerts a critical role in learning and memory processing and was suggested to contribute to the cardinal AD symptom of short-term memory loss [8]. Cortical thinning of grey matter is most pronounced in the entorhinal cortex [9, 10], aligning with neuropathological evidence that indicates the earliest and most significant neurodegenerative changes originate in the entorhinal cortex before spreading to the hippocampus [11]. Notably, BDNF administration to the entorhinal cortex has been shown to mitigate synapse loss, normalize gene expression, enhance neuronal signalling, and improve learning and memory in AD animal models, including mice, aged rats, and monkeys [12].

A functional single nucleotide polymorphism (SNP) in the human BDNF gene, resulting in a valine (Val) to methionine (Met) substitution in the 5′ pro-region of the human BDNF protein, has been associated with the altered activity-dependent secretion of BDNF and hippocampal function [13]. The effects of the BDNF Met allele on various neurological and neurodegenerative disorders including Parkinson’s disease, AD, multiple sclerosis, glaucoma and neuromyelitis optica have been reported and reviewed previously [14, 15]. Consistent with adult findings, reduced cortical thickness was found in the left anterior temporal lobe/entorhinal cortex - regions supporting declarative memory systems in young children [16]. In Met-carrier individuals, a significant genotype-diagnosis interaction revealed reduced volume of the left middle frontal gyrus in major depressive disorder patients compared to healthy subjects, implicating the BDNF Val66Met polymorphism in the early pathogenesis of the disease through prefrontal cortex atrophy [17]. A functional MRI study revealed decreased activation in the cingulate cortex during retrieval tasks in Val homozygotes at high genetic risk for schizophrenia [18]. Further, the BDNF Val66Met polymorphism is significantly correlated with variations in retinal thickness, indicative of its role in the pathophysiology of ocular diseases such as glaucoma and neuromyelitis optica spectrum disorders [19, 20].

In our study with healthy older volunteers, we investigated the impact of the BDNF Val66Met polymorphism on brain structure, diffusion, cognitive performance, and visual functions, focusing on how this genetic variation potentially influences age-related changes in cortical morphology. Beyond traditional volumetric and cortical thickness metrics that target grey matter and atrophy, innovative diffusion indices like Peak Width of Skeletonized Mean Diffusivity (PSMD) and Difference in Distribution Functions (DDF) have been integrated to evaluate the integrity of cerebral white matter. We hypothesized that individuals possessing the BDNF Met allele would demonstrate specific variations in cortical structural metrics, age-associated cognitive functions and retinal measurements when contrasted with Val/Val homozygotes.

## MATERIALS AND METHODS

### Participants

Healthy elderly participants were recruited from the Optic Nerve Decline and Cognitive Change (ONDCC) study between July 2020 and August 2021 at Macquarie University Hospital Ophthalmology Clinic, Sydney, NSW, Australia [21]. All participants included were: (1) of both sexes and above the age of 50 years; (2) able to communicate in English and provide written informed consent; and (3) complete self-report questionnaires and psychometric assessment. The exclusion criteria comprised: (1) a significant history of any type of vision disorders including macular degeneration and glaucoma; (2) ocular surgery within the past two months; (3) diagnosis of dementia, depression, neurotrauma, progressive malignancy, or any other major neurological disease; (4) intellectual disability. Details on the enrolment, composition and selective attrition are described previously [21]. Blood samples were also collected from all study participants.

All procedures used in the current study adhered to the tenets of the Declaration of Helsinki and were approved by the Human Research Ethics Committee Review Board of Macquarie University (5201955 3810900, Sydney, NSW, Australia). Written informed consent was obtained from all study participants.

### Magnetic resonance imaging (MRI) brain imaging

All study participants were invited to undertake an MRI scan, and those who agreed were screened for contraindications (including pacemaker, metallic implant or foreign bodies, cochlear implants, ferromagnetic homeostatic clips, and claustrophobia) and were then scanned using a 3.0T GE DiscoveryTM MR750w Wide Bore MRI scanner (GE Healthcare, Milwaukee, WI, USA) located at the Macquarie Medical Imaging, Sydney, NSW, Australia. Details of the sequence have been described previously [21]. T1-weighted images were obtained using a Magnetization-prepared Rapid Acquisition Gradient Echo (MPRAGE) sequence with prospective motion correction (PROMO). Key scanning parameters included: repetition time (TR) = 8.388 ms, echo time (TE) = 3.168 ms, inversion time (TI) = 900 ms, flip angle (FA) = 8 degrees, and pixel bandwidth = 244.141 Hz. The acquisition matrix = 256×256, with 198 slices, producing 1 mm isotropic voxels. Parallel imaging was facilitated using Auto-calibrating Reconstruction for Cartesian imaging (ARC) with an acceleration factor of 3 in the phase encoding direction.

### Quantification of brain measurements and quality control

Whole brain volumetric analysis was performed using T1-weighted MPRAGE scans that were processed with the standard FreeSurfer pipeline (version 7.1.0 [22]). The assessment included volumetric and/or thickness analyses of the following brain regions: Estimated total intracranial volume (eTIV), Total gray matter (GM), Cortex, Cerebral white matter (WM), Entorhinal cortex, Para-hippocampal cortex, Fusiform cortex, Inferior temporal gyrus, Posterior-cingulate cortex, Cerebellum cortex, Thalamus, Caudate, Putamen, Pallidum, Hippocampus, Amygdala, GM cingulate, GM Parietal lobe, Third ventricle, Forth ventricle and WM hypointensity. These parameters were selected as they have been identified in various studies and reviews as correlates of age-related neurodegeneration.

Quantitative measures including Skeletonized Axial Diffusivity (AD), Fractional Anisotropy (FA), Mean Diffusivity (MD), Mode of Anisotropy (MO), Radial Diffusivity (RD), PSMD and DDF were also processed and derived from diffusion tensor imaging (DTI) scans as previously described [23, 24]. Diffusion metrics such as skeletonised AD, FA, MD, and RD were computed using Tract-Based Spatial Statistics (TBSS) within the FSL, a widely utilized public domain neuroimaging analysis package [25, 26]. Additionally, novel diffusion-related white matter measures such as PSMD and DDF have been implemented. Introduced in 2016, PSMD quantifies cerebral small vessel disease (CSVD) by measuring the range between the 95th and 5th percentiles of skeletonized MD [23]. DDF was developed to characterize the white matter integrity [24]. Diffusion imaging was employed to examine cerebral white matter integrity, complementing the grey matter and atrophy analyses predominately derived from FreeSurfer outputs.

The processing involved excising non-brain tissue, transforming to standard space, segmenting subcortical and deep grey matter structures, standardizing signal intensity, tessellation of the boundary between grey and white matter, applying automated topological correction, and reconstructing grey-white and grey-cerebrospinal fluid surfaces. Volumetric quantification of cortical and subcortical entities was conducted subsequently. Adherence to the ENIGMA Cortical Quality Control Protocol 2.0 ensured the integrity of FreeSurfer outcomes, incorporating both internal and external evaluative imaging. The number of removed values for each measure ranged from 0 to 15 (left banks of the superior temporal sulcus and right pericalcarine regions had 15 poor-quality observations).

### OCT and OCTA acquisition and processing

All subjects underwent optical coherence tomography (OCT) peripapillary circular scans at approximately 3.4 mm diameter for retinal nerve fibre layer (RNFL) thickness measures and macular posterior pole scan for ganglion cell-inner plexiform layer (GCIPL) thickness (Spectralis HRA + OCT; Heidelberg Engineering, Heidelberg, Germany, Version 4.0) [27]. Scans with a quality score of <15 were excluded from the analysis as recommended by the Heidelberg guideline. OCT angiography (OCTA) was acquired to assess the macular superficial vascular complex (SVC; ILM, IPL-) and deep capillary plexus (DCP; IPL+, OPL). Images were excluded if the OCTA quality was < 27.

Scans were assessed and automatic segmentation of the RNFL, GCL and IPL were reviewed by two assessors (TS and SS) for OCT and by (AS and DS) for OCTA, and poor-quality images or those with artefacts were excluded from statistical analysis. An internal 6 × 6 grid was analysed from the posterior pole macular thickness map, as described previously [27, 28]. The vessel density (VD) and vascular feature analyses were conducted utilising ImageJ software (National Institutes of Health, available at https://imagej.net/Fiji/Downloads) with a method adapted from Elfarnawany et al [21, 29, 30]. In brief, the macula OCTA image was binarized and skeletonized in the ImageJ. The foveal avascular zone (FAZ) features were enhanced using magnification, and the FAZ border was delineated manually with the polygon selection tool. Quantitative measures such as area (mm²), perimeter (mm), and circularity—a shape index where values closer to 1 denote circularity and values near 0 suggest irregularity—were determined using shape descriptor settings. The FAZ was manually traced, and VD was calculated across a 6 × 6 mm area of the macula, excluding the central foveal region (0.5 mm radius). The OCTA analysis was conducted in a double-blinded manner to control for potential bias, ensuring the evaluators were unaware of participants' BDNF Val66Met genotype, clinical data, and MRI results. The manual scans were also independently verified by two researchers prior to data export.

### Multifocal visual evoked potentials (mfVEPs) acquisition

The multifocal visual evoked potential (mfVEP) is a functional evaluation that provides a comprehensive topographic assessment of the visual pathways. It reflects the number of functional optic nerve fibers and identifies abnormalities within the optic nerve and associated visual processing regions [31]. MfVEP was conducted using the Vision Search 1 perimetry system controlled by Terra software 1.0 (VisionSearch, Sydney, Australia) with standard stimulus conditions (VisionSearch, Sydney, Australia) as previously reported [32]. Amplitude and latency values were averaged from each segment and used for mfVEP analysis.

### Neuropsychological tests

Several cognitive domains were assessed using a neuropsychological test battery, which comprised: (1) the Mini-Mental Status Exam (MMSE) [33], (2) the national adult reading test (NART) [34], (3) clinical dementia rating (CDR) [35], (4) 15-item Geriatric Depression Scale [36], (5) Logical Memory subtest from the Wechsler Memory Scale (WMS); story A only [37], (6) California Verbal Learning Test, second edition (CVLT-II) [38], (7) Digit Span [39], (8) Trail Making Test (TMT) Part A and B [40], (9) FAS letter fluency [41], (10) Rey complex figure test (RCFT) [42], (11) symbol digit modalities test (SDMT) [43], (12) 30-item Boston naming test (BNT) [44], (13) category fluency (animal naming) [41]. The premorbid Predicted Wechsler Adult Intelligence Scale-Fourth Edition (WAIS-IV) full-scale IQ was calculated using the equation as follows: 126.41 - 0.9775 × NART errors [45].

### Genotyping

The genomic DNA was isolated from peripheral blood utilizing a commercially available DNA extraction kit (Qiagen, Hilden, Germany). Extracted DNA was then quantified with a spectrophotometer (Thermo Scientific, Rockford, IL, USA). The G to A nucleotide substitution, identifying the BDNF Val66Met variant, was amplified by polymerase chain reaction (PCR, Eppendorf, Hamburg, Germany) as previously described [20, 46] with the manufacturer’s protocol. The primers used for PCR were forward 5’ ACTCTGGAGAGCGTGAATGG 3’ and reverse 5’ TCCAGGGTGATGCTCAGTAGT 3’. The genotypes of BDNF Val66Met were determined by direct sequencing of PCR products (both directions, Australian Genome Research Facility, Sydney, Australia).

**Table 1:** Demographic and clinical characteristics of healthy aging participants.

Demographic Data		All	Val/Val	Met carriers
No.	Total, No. (%)	108	64 (59.3)	44 (10.7)
	No. with MRI, No. (%)	81	49 (60.5)	32 (39.5)
Age, yrs		67.3 (9.1)	67.9 (9.1)	66.5 (9.0)
Female, No. (%)		56 (51.9)	37 (57.8)	19 (43.1)
OCT	Global RNFL, μm	96.5 (9.2)	96.3 (9.1)	96.8 (9.6)
	Temporal RNFL, μm	70.8 (11.7)	69.2 (9.8)	73.1 (13.7)
	Nasal RNFL, μm	77.1 (12.2)	76.6 (11.2)	77.8 (13.6)
	Superior RNFL, μm	119.5 (14.4)	119.2 (13.8)	120.0 (15.4)
	Inferior RNFL, μm	129.0 (18.0)	130.9 (17.1)	126.5 (19.1)
	Macular temporal p-pole, μm	66.7 (5.4)	66.4 (5.3)	67.1 (5.5)
OCT Angiography	VD SCP, %	23.3 (5.5)	24.2 (5.9)	22.7 (3.9)
	VD DCP, %	20.9 (7.5)	21.3 (8.9)	20.4 (5.1)
	Superficial FAZ area, mm2	0.4 (0.2)	0.365 (0.2)	0.398 (0.1)
	Deep FAZ area, mm2	0.4 (0.1)	0.355 (0.2)	0.395 (0.1)
mfVEP	Amplitude, μV	143.6 (48.9)	150.6 (46.2)	132.7 (51.5)
	Latency, ms	142.6 (10.3)	143.1 (10.6)	141.9 (10.1)
Cognitive tests	MMSE	28.4 (2.1)	28.5 (2.0)	28.3 (2.3)
	GDS	1.8 (2.5)	1.7 (2.4)	1.9 (2.6)
	Premorbid Predicted IQ	112.2 (8.9)	113.1 (8.0)	110.9 (10.1)
	TMT-Part A, ms	32.7 (10.3)	31.7 (10.1)	34.2 (10.6)
	TMT-Part B, ms	82.2 (49.5)	75.6 (47.0)	91.9 (52.1)
MRI	eTIV	1564840.5 (174505.0)	1555286.7 (184663.6)	1579633.4 (159306.8)
	Total GM Vol	600370.8 (58853.3)	598332.5 (61022.9)	603526.8 (56167.3)
	Cortex Vol	438564.4 (46790.3)	436555.6 (48205.5)	441674.7 (45113.6)
	Cerebral WM Vol	447269.0 (66108.9)	443860.5 (67564.1)	452546.7 (64528.7)

Data are presented in mean (SD) or N (%). Abbreviations: DCP = deep capillary plexus; eTIV = Estimated total intracranial volume; FAZ = Foveal Avascular Zone; GDS = Geriatric depression scale; GM = Gray matter; Met = Methionine; mfVEP = Multifocal visual evoked potential (mfVEP); MMSE = Mini-mental state examination; MRI = magnetic resonance imaging; p-pole = posterior pole; RNFL = Retinal nerve fiber layer; SCP = superior capillary plexus; TMT = Trail making test; Val = valine; VD = Vessel Density; Vol = Volume; WM = White matter.

### Statistics

Statistical analyses were carried out using SPSS software version 29 (SPSS, Inc., Chicago, IL, United States). The sample size for this observational study was determined empirically by referencing prior literature. Descriptive statistics were calculated as mean (SD) or n (%). Demographic characteristics of the two genotype groups were analysed by Mann-Whitney U tests for continuous variables and Chi-square tests for categorical variables. The average values of the visual parameters from both eyes were calculated and compared across the two genotype groups (Val/Val and Met carriers including both Met/Met and Val/Met). An initial one-sided Student's t-test was performed to identify variables with p-values ≤ 0.2 for further analysis. Subsequently, for each dependent variable, a multivariable linear regression model was constructed. Each model assesses the impact of genotype group on the dependent variables, while adjusting for confounders including age, gender, and Premorbid predicted IQ for all neuropsychological test results, and total intracranial volume for all MRI metrics [47]. This analytical approach ensures that the associations between genotype groups and the outcomes are evaluated considering potential confounding factors. Neuropsychological test results were analyzed using raw scores. Missing data were not imputed in the present study. Significance was established at p < 0.05.

**Table 2:** Differences between BDNF Val/Val genotype and Met carriers for MRI structural measurements in healthy aging subjects using multivariable regression analyses.

Dependent variable^2^	Mean^1^		Genotype group	
	Val/Val	Met carriers	Coefficient (95% CI)	P values
Entorhinal Thickness	6.3	6.2	-0.1 (-0.3-0.08)	0.2
Entorhinal Vol	3900.2	3639.8	-323.9 (-595.8-52.0)	0.02*
Para-hippocampal Thickness	5.4	5.2	-0.2 (-0.4-0.02)	0.07
Inferior-temporal Thickness	5.3	5.2	-0.06 (-0.2-0.06)	0.3
Posterior-cingulate Thickness	4.5	4.5	-0.06 (-0.1-0.03)	0.2
Posterior-cingulate Vol	2855.4	2762.7	-167.1 (-319.8-14.4)	0.03*
Caudate Vol	6580.5	6747.2	98.5 (-292.0-489.0)	0.6
Putamen Vol	8977.6	9203.4	130.1 (-406.5-666.7)	0.6
Hippocampus Vol	7986.3	7806.7	-246.7 (-568.7-75.4)	0.1
WM hypointensity	3081.7	4308.7	1336.5 (-627.3-3300.3)	0.2
Skeletonized FA	0.5	0.5	-0.002 (-0.01-0.006)	0.6
Skeletonized MO	0.4	0.4	-0.003 (-0.009-0.004)	0.4
DDF	1.0	1.0	-0.004 (-0.012-0.004)	0.3

1 Mean of each dependent variable.

2 For each dependent variable, a multivariable linear regression model was constructed. Each model assesses the impact of genotype group on the dependent variables, while adjusting for confounders including age, gender, and estimated total intracranial volume (eTIV). This analytical approach ensures that the associations between genotype groups and the outcomes are evaluated considering potential confounding factors. Abbreviations: DDF = Diffusion Degree of Freedom; FA = Fractional Anisotropy; MO = Mode of Anisotropy; Vol = Volume; WM = White matter; Borderline statistical significance: P < 0.1; *P < 0.05.

## RESULTS

**Figure 1.**
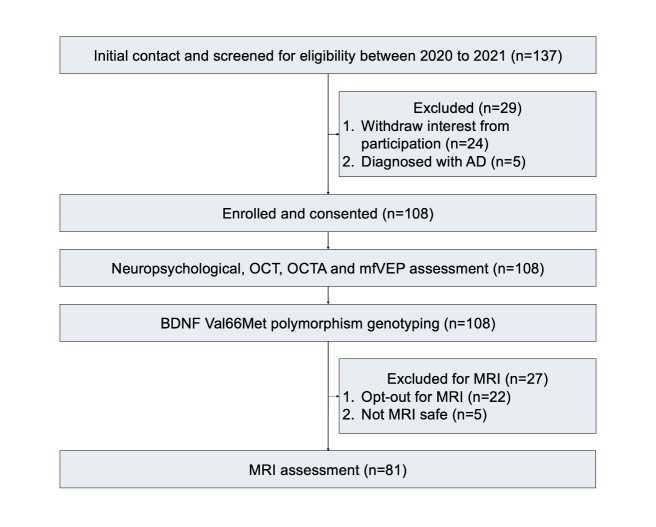
Diagram showing study design. Abbreviations: AD = Alzheimer’s disease; OCT = optical coherence tomography; OCTA = optical coherence tomography angiography; mfVEP = multifocal visual evoked potentials; MRI = magnetic resonance imaging.

Following initial eligibility screening of 137 subjects, 108 were successfully enrolled in the ONDCC study. These 108 individuals comprised the entire cohort at the start of our current study and were all included in this research. The study design is presented as a flow diagram (Fig. 1), all participants underwent genotyping for BDNF Val66Met polymorphism status, OCT, OCTA and mfVEP, and 81 participants had MRI scans (Figure 1). This study included 56 women and 52 men, with demographic details as well as ophthalmic, neuropsychological and MRI variables summarised in Table 1 for all participants. The mean (SD) age of study participants was 67.3 (9.1) years. There were no significant differences between groups in gender (χ^2^ = 2.2, *P* = 0.1) and age (*P* = 0.7) distribution. The BDNF Val66Met genotype distribution among the study cohort was 64 (59.3%) for genotype GG (Val/Val), 39 (36.1%) for GA (Val/Met), and 5 (4.6%) for AA (Met/Met). The allele frequency of the A allele (Met) was 26.5% in the healthy aging subjects. The genotype distribution in our cohort (40.7% in total for Met/Met and Val/Met genotypes) is consistent with that reported in aging studies from the US and Germany, with Met carrier frequencies of 40% and 39.7%, respectively [48, 49].

The BDNF Met carriers were associated with a significantly lower entorhinal cortex volume compared to Val homozygotes (*P* = 0.02), after controlling for confounding factors including age, sex and estimated total intracranial volumes (Table 2). The results from the preliminary analysis are shown in Supplementary Tables 1 and 2. The mean entorhinal volume was lower by 6.7% in the Met carriers’ group compared to the Val/Val group. Statistically significant differences were found between the two genotype groups for posterior cingulate cortex volume (*P* = 0.03) with Met carriers having a 3.2% lower volume compared to Val homozygotes. A borderline significant difference was also found in parahippocampal thickness between the two genotype groups (*P* = 0.07). Other volumetric or thickness measurements as well as diffusion tensor imaging measures were not associated with BDNF Val66Met polymorphism.

Multivariable regression analysis assessing the effect of BDNF polymorphism on a range of ophthalmological parameters and cognitive task measures is presented in Table 3. There was a statistically significant difference between the two genotype groups for temporal RNFL thickness (*P* = 0.04). The carriage of the BDNF Met allele was also associated with a trend of borderline significance of higher foveal avascular zone circularity index deep capillary plexus (*P* = 0.06) and lower logical memory recognition scores (*P* = 0.09).

**Figure 2.**
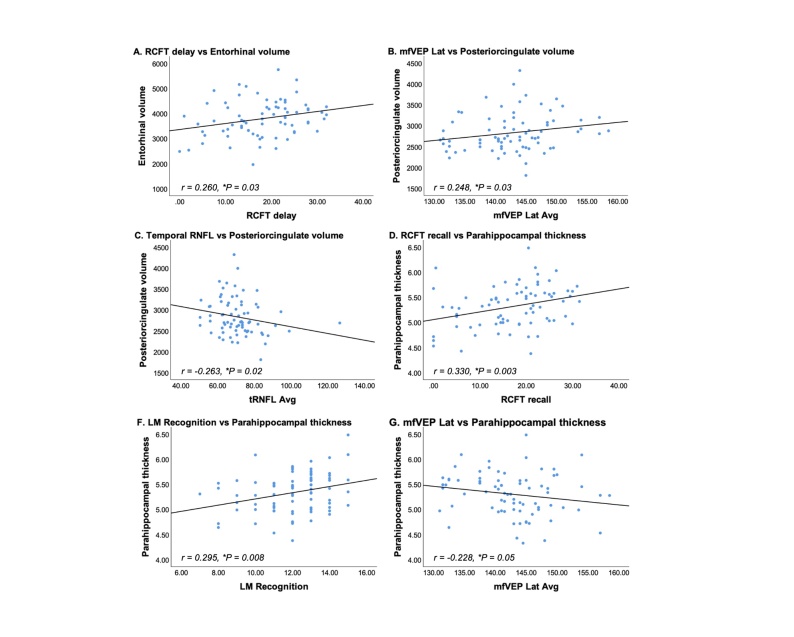
Correlation analyses between morphological brain metrics and visual/neuropsychological parameters in healthy aging cohort. Abbreviations: Avg = average; Lat = Latency; LM = Logical memory; mfVEP = Multifocal visual evoked potential; RCFT = Rey complex figure test; RNFL = Retinal nerve fibre layer; t = temporal. *P < 0.05.

Correlation analyses were performed between cortical morphological measures that were found to be statistically or borderline significantly associated with BDNF Val66Met carriage and vision tests as well as neuropsychological testing scores (Fig. 2). Small but significant correlations were found between entorhinal volume, posterior cingulate cortex volume and parahippocampal thickness with various retinal parameters and cognitive test scores (r ranged from -0.263 to 0.330, *P* < 0.05).

## DISCUSSION

In the present study, we observed for the first time a significant association between BDNF Met variants and lower volumes in the entorhinal and posterior cingulate cortices among a cohort of elderly normal subjects without evidence of dementia. These findings align with prior research, reinforcing the role of BDNF as a genetic contributor to lower volumes in these regions [50]. Additionally, our results indicate no significant differences between the BDNF Val66Met polymorphism and structural brain changes in other regions, cognitive function, or visual and retinal vascular measures except for temporal RNFL thickness. Moreover, these results extend the influence of the BDNF Val66Met polymorphism beyond pathological states, indicating its potential to adversely impact specific brain regions in elderly individuals under normal physiological conditions. This effect is notably more pronounced in memory-associated brain regions compared to overall cognitive function and visual system metrics.

**Table 3:** Differences between BDNF Val/Val genotype and Met carriers for visual structural, functional and neuropsychological measurements in healthy aging subjects using multivariable regression analyses.

Parameters		Mean^1^		Genotype group	
		Val/Val	Met carriers	Coefficient (95% CI)	P-values
OCT	Temporal RNFL	69.2	73.1	4.8 (0.2-9.4)	0.04*
	Temporal superior RNFL	129.3	132.8	4.1 (-4.3-12.5)	0.3
	Temporal inferior RNFL	147.3	143.0	-3.5 (-11.4-4.4)	0.4
	Nasal inferior RNFL	114.2	110.0	-4.3 (-13.1-4.4)	0.3
	Inferior RNFL	130.9	126.5	-4.1 (-11.1-3.0)	0.3
	Temporal superior p-pole	66.7	67.6	0.7 (-1.2-2.6)	0.5
OCT Angiography	VD SCP, %	24.1	22.2	-1.7 (-5.0-1.6)	0.3
	Superficial FAZ area, mm^2^	0.4	0.4	0.04 (-0.06-0.1)	0.5
	Deep FAZ area, mm^2^	0.4	0.4	0.04 (-0.05-0.1)	0.4
	FAZ perimeter SCP, mm	2.2	2.3	0.2 (-0.2-0.5)	0.4
	FAZ perimeter DCP, mm	2.2	2.3	0.1 (-0.1-0.4)	0.3
	FAZ circularity index DCP	0.9	0.9	0.03 (-0.006-0.06)	0.06
mfVEP	Amplitude	150.6	132.7	-14.8 (-34.5-5.0)	0.1
Neuropsychological tests	Premorbid predicted IQ	113.1	110.9	-2.4 (-6.0-1.3)	0.2
	LM-Immediate	13.4	12.6	-0.4 (-2.1-1.2)	0.6
	LM-Delay	11.9	11.0	-0.6 (-2.4-1.2)	0.5
	LM-Recognition	12.3	11.6	-0.6 (-1.3-0.1)	0.09
	CVLT-TL	42.8	40.6	-0.3 (-5.1-4.5)	0.9
	CVLT-sdfr	9.2	8.5	-0.1 (-1.6-1.4)	0.9
	CVLT-ldfr	9.8	8.8	-0.5 (-2.0-1.0)	0.5
	F	15.2	13.8	-0.8 (-2.9-1.3)	0.5
	A	13.2	11.9	-0.4 (-2.2-1.3)	0.6
	S	15.6	14.3	-0.3 (-2.5-1.8)	0.8
	Letter Fluency	44.0	40.1	-1.6 (-6.8-3.6)	0.5
	Category Fluency	20.3	18.7	-1.0 (-2.7-0.7)	0.3
	SDMT	49.2	47.6	-0.9 (-5.1-3.3)	0.7
	DS-Total	28.4	27.6	0.04 (-2.0-2.1)	0.9
	TMT-Part A, s	31.7	34.2	2.7 (-0.9-6.2)	0.1
	TMT-Part B, s	75.6	91.9	15.5 (-3.8-34.8)	0.1
	RCFT-Copy	33.5	34.1	1.1 (-1.9-4.0)	0.5

1 Mean of each dependent variable.

2 For each dependent variable, a multivariable linear regression model was constructed. Each model assesses the impact of genotype group on the dependent variables, while adjusting for confounders including age, gender, and Premorbid predicted IQ for all neuropsychological test results. This analytical approach ensures that the associations between genotype groups and the outcomes are evaluated considering potential confounding factors. Abbreviations: CVLT = California verbal learning test; DCP = Deep capillary plexus; DS = Digit span; FAZ = Foveal Avascular Zone; ldfr = long-delay free recall; LM = Logical memory; mfVEP = Multifocal visual evoked potential; OCT = Optical coherence tomography; p-pole = posterior pole; RCFT = Rey complex figure test; RNFL = Retinal nerve fibre layer; sdfr = short-delay free recall; SDMT = Symbol digit modality test; SCP = Superficial capillary plexus; TL = Total learning; TMT = Trail making test; VD = Vessel Density; Borderline statistical significance: P < 0.1; *P < 0.05.

Multiple lines of evidence have implicated BDNF in the aging and AD processes [51]. BDNF is reported to influence the translation of the activity signal into synaptic plasticity changes and age-related decline in hippocampal volume [52, 53]. In early-stage AD, dose-dependent effects were found between BDNF Val66Met polymorphism and the default mode network in the medial temporal lobe [54]. In a longitudinal study, individuals with both BDNF Met and APOE E4 alleles exhibited more pronounced entorhinal cortex atrophy and cognitive decline over three years compared to Val/Val homozygotes in cases of mild cognitive impairment (MCI) and AD [55]. The concurrent presence of APOE ɛ4 and BDNF polymorphisms were also found to be correlated with worse memory performance in immediate and delayed recalls in patients with amnestic MCI (aMCI) [56]. The entorhinal delivery of the BDNF gene after AD onset reversed neuronal atrophy, improved cognitive decline restored cell signalling and demonstrated substantial neuroprotective effects of BDNF on critical neuronal circuitry in AD models [12]. Our study corroborates these trends, demonstrating lower mean entorhinal volume in Met carriers, consistent with longitudinal studies linking Met/Met and Val/Met subjects with greater entorhinal volume loss and lower MMSE scores than those with the Val/Val genotype [57]. In contrast, Voineskos et al. have reported that the BDNF Val66Met variants interact with age to predict cortical thickness, especially in the entorhinal region, white matter integrity and episodic memory. Specifically, Val homozygotes were susceptible in the late-life while Met carriers were more vulnerable in early adult life [58]. Our results did not show significant differences in diffusion tensor imaging parameters between genotypes. The contrasting findings are likely attributed to age differences among cohorts—our healthy aging cohort's mean age is 67 years, younger than the cohort studied by Xia et al. (73.8 years) but older than that studied by Voineskos et al. (46 years).

Research has linked BDNF signaling and Val66Met polymorphisms to functional alterations in the anterior but not posterior cingulate cortex across major depressive disorders as well as normative conditions [59-61]. Given the established connection between posterior cingulate cortex dysfunction and deficits in episodic memory tasks in mild cognitive impairment [62, 63], our findings of lower volume in this region may indicate a detrimental impact of Met allele carriage on the healthy aging brain. This observation is further substantiated by a trend towards lower recognition scores in the logical memory test, possibly indicative of compromised episodic memory.

The BDNF variation did not show a significant association with cognitive functions which are prominently affected in healthy aging. Nonetheless, there was a discernible trend indicating diminished performance in logical memory recognition and TMT Part B among Met allele carriers, hinting at BDNF Met's possible association with memory and executive function. Significant differences in temporal RNFL thickness were noted between two genotype groups, with Met carriers exhibiting thicker temporal RNFL, yet no significant variances were observed in other ophthalmologic measures. Interestingly, this aligns with prior findings that Val homozygotes, particularly in a cohort of open-angle glaucoma patients aged around 70 years, showed a significant association with accelerated progression marked by global RNFL deterioration [20]. Given the comparable mean age of our elderly normal subjects without evidence of dementia to the glaucoma group, it suggests potential neuroprotective roles of BDNF Val66Met on retinal morphology under physiological conditions, especially regarding the temporal RNFL in older adults. However, RNFL thickness variability may also arise from inter-individual differences, including age, sex, ethnicity [64], optic disc size, foveal structure, retinal vessel arrangement, axial length, and refractive errors [65-67], complicating standardization.

Evidence suggests that deficits in long-term potentiation and spatial learning in aging may occur independently of neuron loss and are correlated with reduced BDNF levels or alterations in BDNF-related signalling pathways [68]. BDNF overexpression in transgenic mouse models has been associated with disrupted normal brain functions, manifesting as learning deficits, heightened seizure severity, and increased hippocampal and entorhinal cortex excitability [69]. The BDNF levels in the entorhinal cortex were significantly lower in AD patients compared to healthy controls [70]. Similarly, post-mortem analysis has indicated that neurons displaying neurofibrillary tangles typically lacked BDNF immunoreactivity, while those with intense BDNF labelling were generally spared from extensive neurofibrillary degeneration [71].

Several advantages and limitations of our study warrant discussion. First, the present study comprised a comprehensive set of parameters including brain volumetric, diffusion and cognitive function parameters, as well as structural and functional measurements of the visual system. The observed lower entorhinal and posterior cingulate volumes associated with BDNF Val66Met polymorphism carriage likely reflect a genuine variance between genotypes. Our sample size was relatively small; however, the sex and genotype distribution were favourable in the cohort. A limitation was the omission of tau pathology analysis, which is suggested to originate in the entorhinal cortex and hippocampus and is linked to cognitive performance and hippocampal connectivity [72, 73]. Additionally, our findings imply a causal relationship; however, the cross-sectional design of this study limits our ability to definitively confirm causality. Specifically, it remains unclear whether the observed disparities between the two genotype groups emerged during brain development or are attributable to age-related decline. To validate the long-term effects of the BDNF Val66Met polymorphism on the aging brain, future longitudinal cohort studies incorporating larger sample sizes and including both brain volumetric and visual system data are essential. Additionally, utilizing animal models with the BDNF Val66Met gene polymorphism will be instrumental in elucidating the mechanisms underlying the genotypic effects on aging and determining if these effects manifest during brain development.

In summary, our study established a significant association between BDNF Met alleles and lower mean volumes in the entorhinal and posterior cingulate cortices in a healthy elderly cohort. The findings imply an adverse impact of the BDNF Met allele on the volumetric integrity of these cortices in the elderly normal subjects without evidence of dementia relative to the naturally occurring Val homozygotes.

## Supplementary Materials

The Supplementary data can be found online at: www.aginganddisease.org/EN/10.14336/AD.2024.0346.

## Data Availability

The original contributions presented in the study are included in the article/supplementary material, further inquiries can be directed to the corresponding author/s.
