# Supplementary material for: Brain-Derived Neurotrophic Factor Val66Met is Associated with Variation in Cortical Structure in Healthy Aging Subjects
Source: Aging Dis. 2024 Oct 1;15(5):2315–27. doi: 10.14336/AD.2024.0346 (PMC11346411; doi:10.14336/AD.2024.0346)
Supplement: Supplementary file 1 — The Supplementary data can be found online at: www.aginganddisease.org/EN/10.14336/AD.2024.0346. [file AD-15-5-2315-s.pdf]

SUPPLEMENTARY DATA

Brain-Derived Neurotrophic Factor Val66Met is Associated with Variation in Cortical Structure in Healthy Aging Subjects

Ting Shen, Samran Sheriff, Yuyi You, Jiyang Jiang, Angela Schulz, Heather Francis, Mehdi Mirzaei, Danit Saks, Viswanthram Palanivel, Devaraj Basavarajappa, Nitin Chitranshi, Veer Gupta, Wei Wen, Perminder S. Sachdev, Huixun Jia, Xiaodong Sun, Stuart L. Graham, Vivek K. Gupta

© 2024. Shen T et al. Published online at http://www.aginganddisease.org/EN/10.14336/AD.2024.0346

SUPPLEMENTARY DATA

Supplementary Table 1. Differences between BDNF Val/Val genotype and Met carriers and MRI structural measurements in healthy aging subjects in preliminary analysis.

	Mean		Genotype	
	Val/Val	Met carriers	Mean Differences (95% CI)	P-values
**eTIV**	1555286.7	1579633.4	-24346.8 (-104741.1-56047.6)	0.3
**Total GM Vol**	598332.5	603526.8	-5194.4 (-32346.4-21957.7)	0.4
**Cortex Vol**	436555.6	441674.7	-5119.1 (-26695.0-16456.7)	0.3
**Cerebral WM Vol**	443860.5	452546.7	-8686.2 (-39150.7-21778.2)	0.3
**Entorhinal Thickness**	6.3	6.2	0.1 (-0.08-0.3)	0.1
**Entorhinal Vol**	3900.2	3639.8	260.4 (-72.0-592.9)	0.06
**Para-hippocampal Thickness**	5.4	5.2	0.2 (0.03-0.4)	0.01
**Para-hippocampal Vol**	4071.5	4005.2	66.4 (-161.3-294.0)	0.3
**Fusiform Thickness**	5.3	5.3	0.04 (-0.08-0.2)	0.3
**Fusiform Vol**	18450.3	18790.7	-340.3 (-1602.4-921.8)	0.3
**Inferior-temporal Thickness**	5.3	5.2	0.07 (-0.05-0.2)	0.1
**Inferior-temporal Vol**	20550.6	20532.0	18.6 (-1483.8-1521.0)	0.5
**Posterior-cingulate Thickness**	4.5	4.5	0.04 (-0.05-0.1)	0.2
**Posterior-cingulate Vol**	2855.4	2762.7	92.7 (-113.5-298.8)	0.2
**Cerebellum Cortex Vol**	107419.2.2	107248.4	170.8 (-5119.8-5461.5)	0.5
**Thalamus Vol**	13300.1	13402.6	-102.6 (-729.5-524.3)	0.4
**Caudate Vol**	6580.5	6747.2	-166.8 (-618.7-285.1)	0.2
**Putamen Vol**	8977.6	9203.4	-225.7 (-812.5-361.1)	0.2
**Pallidum Vol**	3697.9	3690.5	7.4 (-228.0-242.7)	0.5
**Hippocampus Vol**	7986.3	7806.7	179.6 (-246.3-605.5)	0.2
**Amygdala Vol**	3111.9	3172.6	-60.7 (-256.7-135.2)	0.3
**GM Cingulate Vol**	18131.3	18510.4	-379.1 (-1507.1-749.0)	0.3
**GM Parietal Vol**	101858.3	101761.8	96.5 (-5234.0-5427.0)	0.5
**Third Ventricle Vol**	1515.0	1578.1	-63.0 (-316.4-190.3)	0.3
**Forth Ventricle Vol**	1931.6	1935.8	-4.2 (-211.8-203.4)	0.5
**WM hypointensity**	3081.7	4308.7	-1226.9 (-3287.9-834.1)	0.1
**Skeletonized AD**	0.008	0.008	<0.00001 (-0.00001-<0.00001)	0.2
**Skeletonized FA**	0.5	0.5	0.004 (-0.005-0.01)	0.2
**Skeletonized MD**	0.0005	0.0005	<0.00001 (-0.00001-<0.00001)	0.1
**Skeletonized MO**	0.4	0.4	0.004 (-0.003-0.01)	0.1
**Skeletonized RD**	0.003	0.004	<0.00001 (-0.00001-<0.00001)	0.1
**PSMD**	0.002	0.002	-0.00001 (-0.00002-<0.00001)	0.1
**DDF**	1.0	1.0	0.005 (-0.004-0.01)	0.1

Abbreviations: AD = Axial Diffusivity; DDF = Diffusion Degree of Freedom; eTIV = Estimated total intracranial volume; FA = Fractional Anisotropy; GM = Grey matter; MD = Mean Diffusivity; MO = Mode of Anisotropy; PSMD = Peak Width of Skeletonized Mean Diffusivity; RD = Radial Diffusivity; Vol = Volume; WM = White matter; *P < 0.05.

Supplementary Table 2. Differences between BDNF Val/Val genotype and Met carriers and visual structural, functional and neuropsychological measurements in healthy aging subjects in preliminary analysis.

Parameters		Mean		Genotype	
		Val/Val	Met carriers	Mean Differences (95% CI)	P-values
**OCT**	Global RNFL	96.3	96.8	-0.4 (-4.2-3.3)	0.4
	Temporal RNFL	69.2	73.1	-3.9 (-8.6-0.7)	0.05*
	Temporal superior RNFL	129.3	132.8	-3.5 (-11.9-5.0)	0.2
	Temporal inferior RNFL	147.3	143.0	4.3 (-3.9-12.5)	0.2
	Nasal RNFL	76.6	77.8	-1.2 (-6.1-3.6)	0.3

© 2024. Shen T et al. Published online at http://www.aginganddisease.org/EN/10.14336/AD.2024.0346

SUPPLEMENTARY DATA

	Nasal superior RNFL	108.2	107.3	1.0 (-7.6-9.5)	0.4
	Nasal inferior RNFL	114.2	110.0	4.2 (-4.5-12.9)	0.2
	Superior RNFL	119.2	120.0	-0.8 (-6.6-5.0)	0.4
	Inferior RNFL	130.9	126.5	4.4 (-2.8-11.6)	0.1
	Temporal p-pole	66.4	67.1	-0.7 (-2.8-1.4)	0.3
	Temporal superior p-pole	66.7	67.6	-0.9 (-3.1-1.4)	0.2
	Temporal inferior p-pole	66.5	67.0	-0.5 (-2.7-1.8)	0.3
**OCT Angiography**	VD SCP, %	24.1	22.2	-1.5 (-4.7-1.8)	0.2
	VD DCP, %	21.2	20.5	-0.9 (-5.7-3.8)	0.3
	Superficial FAZ area, mm2	0.4	0.4	0.03 (-0.06-0.1)	0.2
	Deep FAZ area, mm2	0.4	0.4	0.04 (-0.05-0.1)	0.2
	FAZ perimeter SCP, mm	2.2	2.3	0.1 (-0.2-0.5)	0.2
	FAZ perimeter DCP, mm	2.2	2.3	0.1 (-0.1-0.4)	0.2
	FAZ circularity index SCP	0.9	0.9	0.01 (-0.02-0.4)	0.5
	FAZ circularity index DCP	0.9	0.9	0.03 (-0.006-0.06)	0.06
**mfVEP**	Amplitude	150.6	132.7	17.9 (-1.8-37.6)	0.04*
	Latency	143.1	141.9	1.3 (-3.0-5.5)	0.3
**Neuropsychological tests**	Premorbid predicted IQ	113.1	110.9	2.1 (-1.4-5.7)	0.1
	LM-Immediate	13.4	12.6	0.7 (-0.9-2.4)	0.2
	LM-Delay	11.9	11.0	1.0 (-0.8-2.8)	0.1
	LM-Recognition	12.3	11.6	0.7 (-0.01-1.4)	0.03*
	LM-Delay/Immediate Percent	88.5	83.3	-5.1 (-14.2-3.9)	0.1
	CVLT-TL	42.8	40.6	2.2 (-3.1-7.6)	0.2
	CVLT-sdfr	9.2	8.5	0.6 (-0.9-2.2)	0.2
	CVLT-ldfr	9.8	8.8	1.0 (-0.6-2.6)	0.1
	F	15.2	13.8	1.4 (-0.8-3.6)	0.1
	A	13.2	11.9	1.3 (-0.6-3.2)	0.09
	S	15.6	14.3	1.2 (-1.1-3.6)	0.2
	Letter Fluency	44.0	40.1	3.9 (-1.8-9.7)	0.09
	Category Fluency	20.3	18.7	1.6 (-0.4-3.5)	0.06
	SDMT	49.2	47.6	1.6 (-2.9-6.1)	0.2
	DS-Total	28.4	27.6	0.8 (-1.4-3.0)	0.2
	TMT-Part A, s	31.7	34.2	-2.5 (-6.7-1.6)	0.1
	TMT-Part B, s	75.6	91.9	-16.4 (-36.4-3.7)	0.05
	BNT-NCS	27.4	27.5	-0.2 (-1.5-1.1)	0.4
	MMSE	28.5	28.3	0.2 (-0.6-1.1)	0.3
	GDS	1.7	1.9	-0.2 (-1.2-0.8)	0.4
	RCFT-Copy	33.5	34.1	-0.6 (-2.3-1.0)	0.2
	RCFT-Immediate recall	17.4	18.0	-0.6 (-3.6-2.5)	0.4
	RCFT-Delayed recall	16.5	17.0	-0.5 (-3.7-2.7)	0.4

Abbreviations: BNT NCS = Boston naming test-no cue score; CVLT = California verbal learning test; DCP = Deep capillary plexus; DS = Digit span; FAZ = Foveal Avascular Zone; GDS = Geriatric depression scale; ldfr = long-delay free recall; LM = Logical memory; mfVEP = Multifocal visual evoked potential; MMSE = Mini-mental state examination; OCT = Optical coherence tomography; p-pole = posterior pole; RCFT = Rey complex figure test; RNFL = Retinal nerve fibre layer; sdfr = short-delay free recall; SCP = Superficial capillary plexus; SDMT = Symbol digit modality test; TL = Total learning; TMT = Trail making test; VD = Vessel Density; *P < 0.05.

© 2024. Shen T et al. Published online at http://www.aginganddisease.org/EN/10.14336/AD.2024.0346
